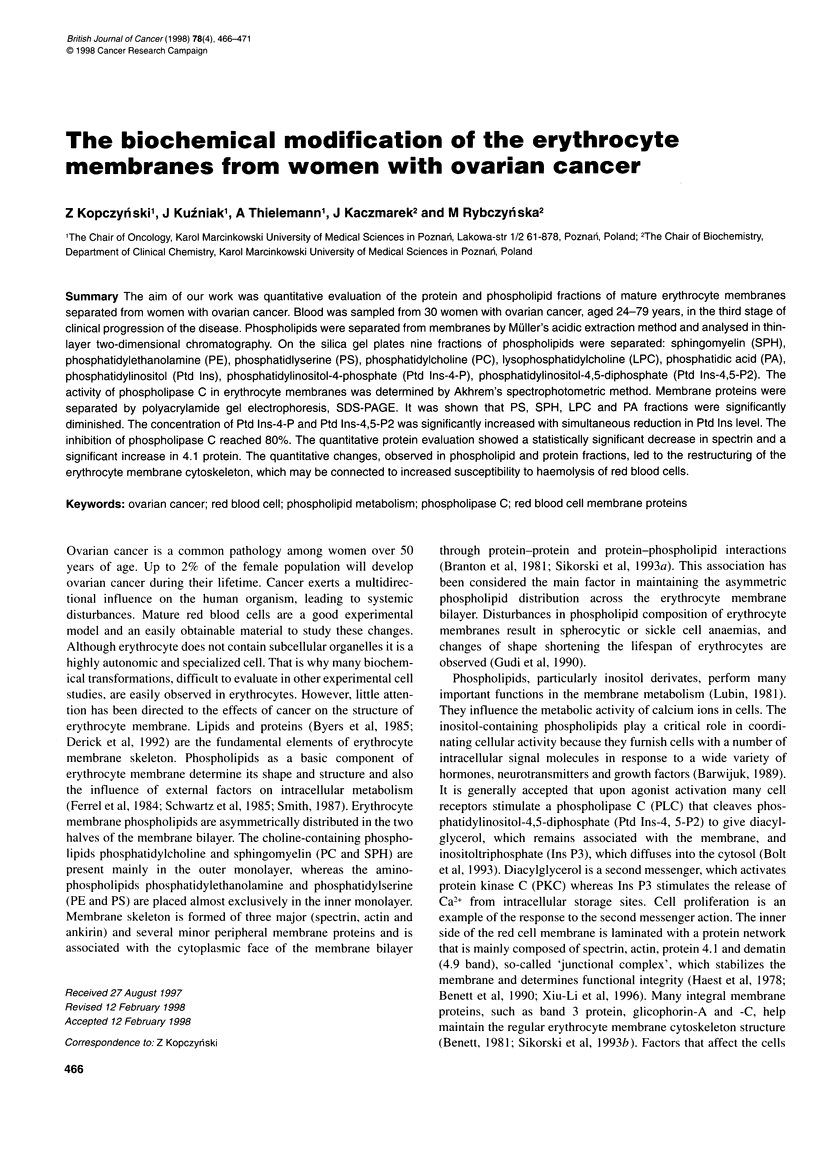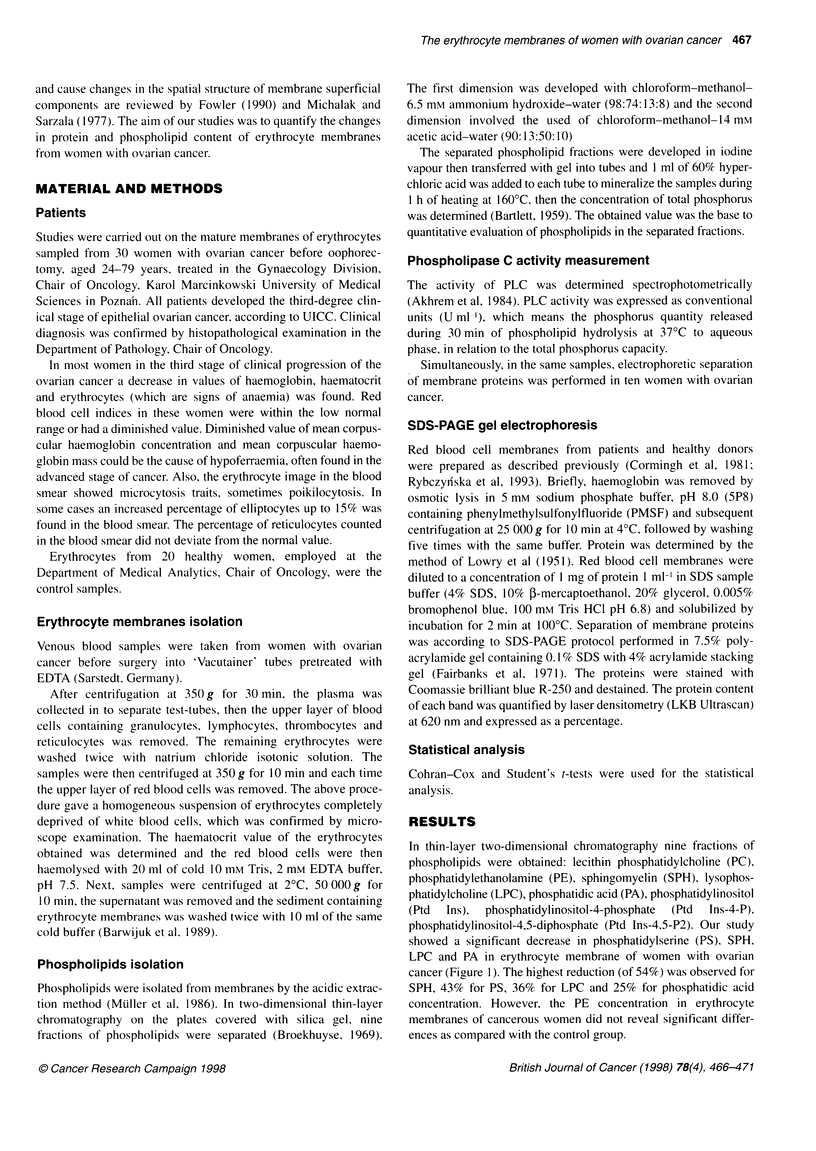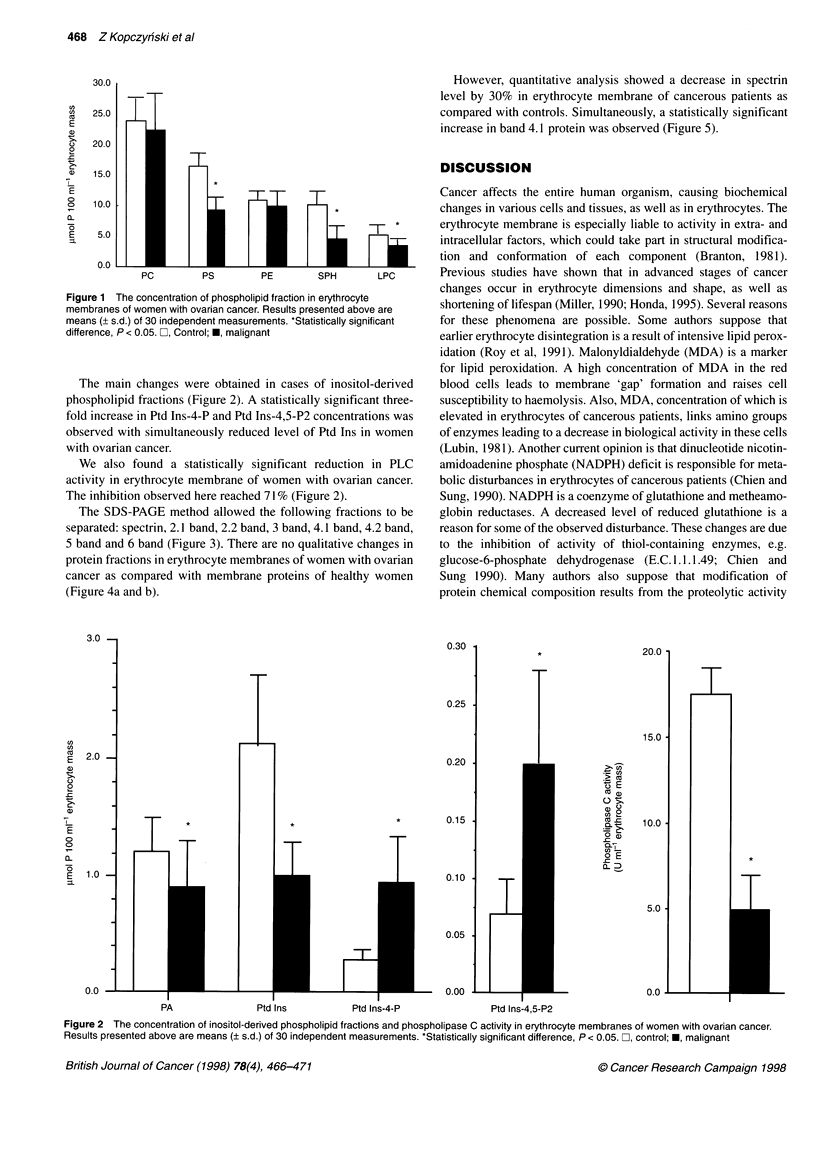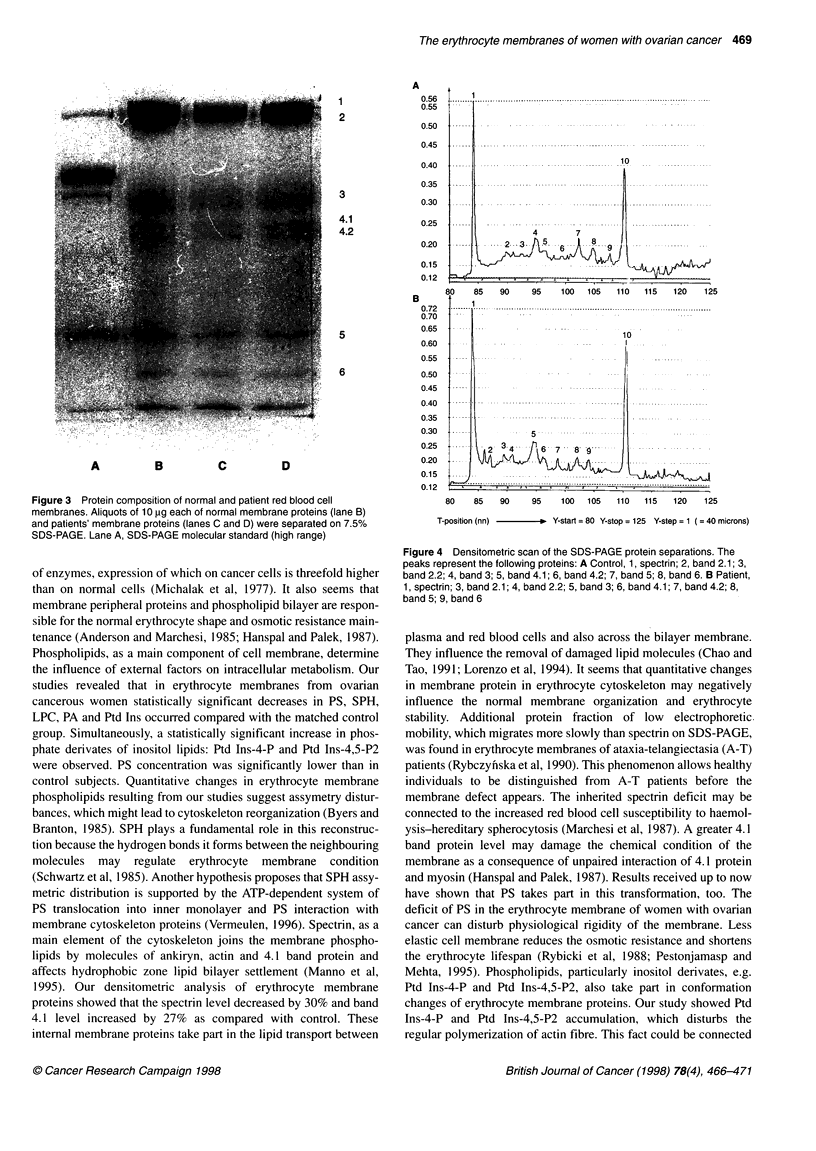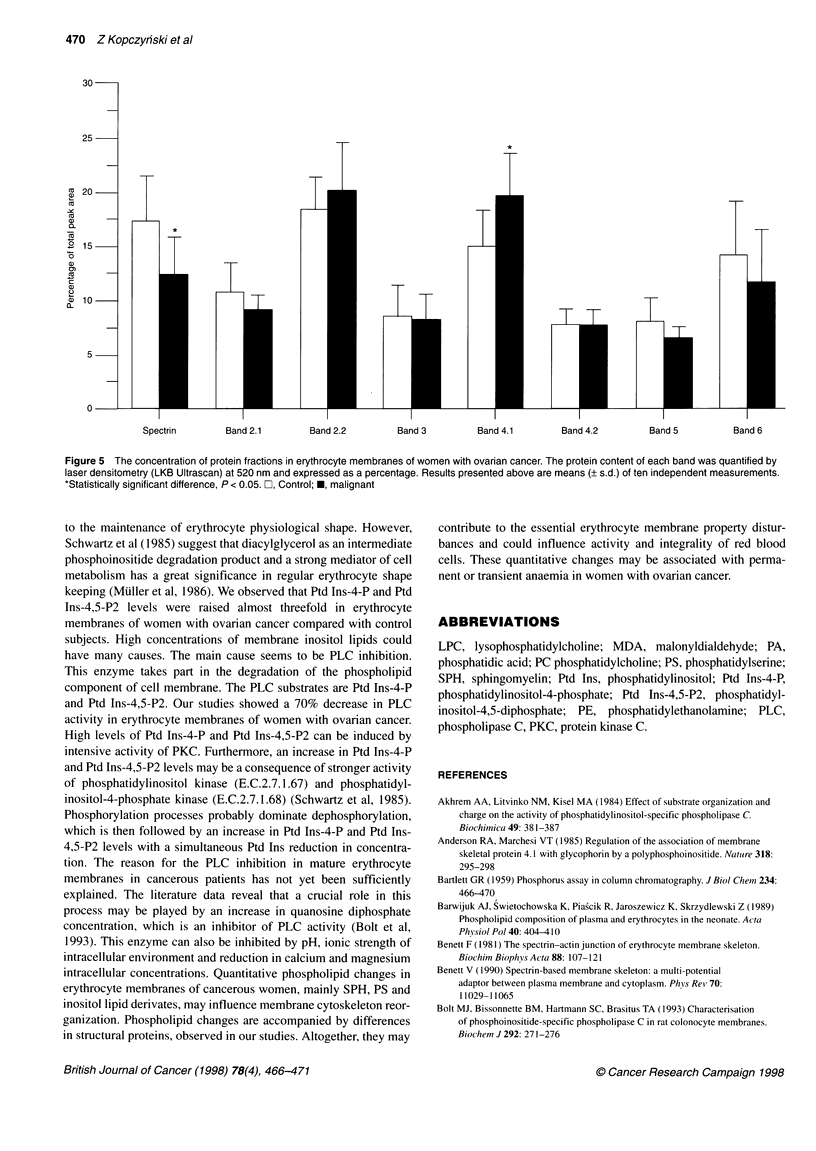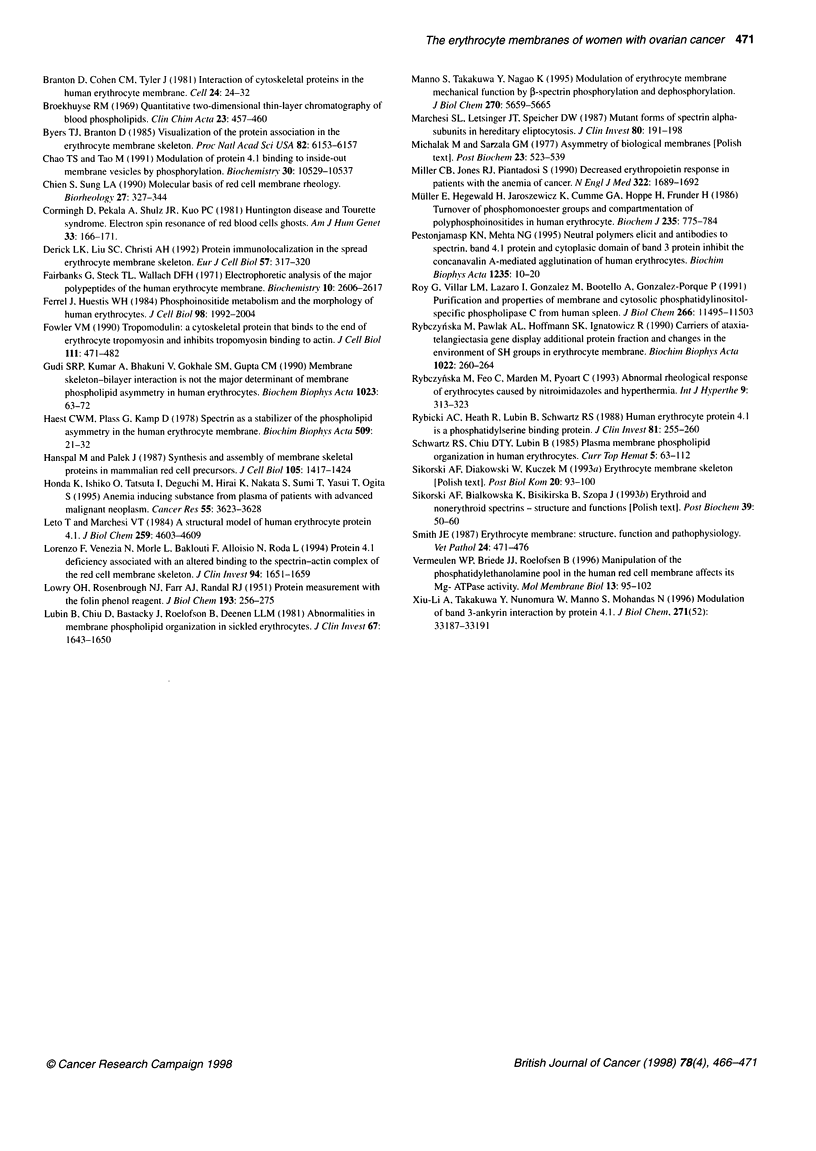# The biochemical modification of the erythrocyte membranes from women with ovarian cancer.

**DOI:** 10.1038/bjc.1998.516

**Published:** 1998-08

**Authors:** Z. Kopczyński, J. Kuźniak, A. Thielemann, J. Kaczmarek, M. Rybczyńska

**Affiliations:** Oncology, Karol Marcinkowski University of Medical Sciences in Poznań, Poland.

## Abstract

**Images:**


British Journal of Cancer (1998) 78(4), 466–471
© 1998 Cancer Research Campaign

### The biochemical modification of the erythrocyte membranes from women with ovarian cancer

**Z Kopczyński[1], J Kuźniak[1], A Thielemann[1], J Kaczmarek[2] and M Rybczyńska[2]**

[1]The Chair of Oncology, Karol Marcinkowski University of Medical Sciences in Poznań, Lakowa-str 1/2 61-878, Poznań, Poland; [2]The Chair of Biochemistry, Department of Clinical Chemistry, Karol Marcinkowski University of Medical Sciences in Poznań, Poland

**Summary** The aim of our work was quantitative evaluation of the protein and phospholipid fractions of mature erythrocyte membranes separated from women with ovarian cancer. Blood was sampled from 30 women with ovarian cancer, aged 24–79 years, in the third stage of clinical progression of the disease. Phospholipids were separated from membranes by Müller's acidic extraction method and analysed in thin-layer two-dimensional chromatography. On the silica gel plates nine fractions of phospholipids were separated: sphingomyelin (SPH), phosphatidylethanolamine (PE), phosphatidlyserine (PS), phosphatidylcholine (PC), lysophosphatidylcholine (LPC), phosphatidic acid (PA), phosphatidylinositol (Ptd Ins), phosphatidylinositol-4-phosphate (Ptd Ins-4-P), phosphatidylinositol-4,5-diphosphate (Ptd Ins-4,5-P2). The activity of phospholipase C in erythrocyte membranes was determined by Akhrem's spectrophotometric method. Membrane proteins were separated by polyacrylamide gel electrophoresis, SDS-PAGE. It was shown that PS, SPH, LPC and PA fractions were significantly diminished. The concentration of Ptd Ins-4-P and Ptd Ins-4,5-P2 was significantly increased with simultaneous reduction in Ptd Ins level. The inhibition of phospholipase C reached 80%. The quantitative protein evaluation showed a statistically significant decrease in spectrin and a significant increase in 4.1 protein. The quantitative changes, observed in phospholipid and protein fractions, led to the restructuring of the erythrocyte membrane cytoskeleton, which may be connected to increased susceptibility to haemolysis of red blood cells.

**Keywords:** ovarian cancer; red blood cell; phospholipid metabolism; phospholipase C; red blood cell membrane proteins

Ovarian cancer is a common pathology among women over 50 years of age. Up to 2% of the female population will develop ovarian cancer during their lifetime. Cancer exerts a multidirectional influence on the human organism, leading to systemic disturbances. Mature red blood cells are a good experimental model and an easily obtainable material to study these changes. Although erythrocyte does not contain subcellular organelles it is a highly autonomic and specialized cell. That is why many biochemical transformations, difficult to evaluate in other experimental cell studies, are easily observed in erythrocytes. However, little attention has been directed to the effects of cancer on the structure of erythrocyte membrane. Lipids and proteins (Byers et al, 1985; Derick et al, 1992) are the fundamental elements of erythrocyte membrane skeleton. Phospholipids as a basic component of erythrocyte membrane determine its shape and structure and also the influence of external factors on intracellular metabolism (Ferrel et al, 1984; Schwartz et al, 1985; Smith, 1987). Erythrocyte membrane phospholipids are asymmetrically distributed in the two halves of the membrane bilayer. The choline-containing phospholipids phosphatidylcholine and sphingomyelin (PC and SPH) are present mainly in the outer monolayer, whereas the aminophospholipids phosphatidylethanolamine and phosphatidylserine (PE and PS) are placed almost exclusively in the inner monolayer. Membrane skeleton is formed of three major (spectrin, actin and ankirin) and several minor peripheral membrane proteins and is associated with the cytoplasmic face of the membrane bilayer through protein–protein and protein–phospholipid interactions (Branton et al, 1981; Sikorski et al, 1993*a*). This association has been considered the main factor in maintaining the asymmetric phospholipid distribution across the erythrocyte membrane bilayer. Disturbances in phospholipid composition of erythrocyte membranes result in spherocytic or sickle cell anaemias, and changes of shape shortening the lifespan of erythrocytes are observed (Gudi et al, 1990).

Phospholipids, particularly inositol derivates, perform many important functions in the membrane metabolism (Lubin, 1981). They influence the metabolic activity of calcium ions in cells. The inositol-containing phospholipids play a critical role in coordinating cellular activity because they furnish cells with a number of intracellular signal molecules in response to a wide variety of hormones, neurotransmitters and growth factors (Barwijuk, 1989). It is generally accepted that upon agonist activation many cell receptors stimulate a phospholipase C (PLC) that cleaves phosphatidylinositol-4,5-diphosphate (Ptd Ins-4, 5-P2) to give diacylglycerol, which remains associated with the membrane, and inositoltriphosphate (Ins P3), which diffuses into the cytosol (Bolt et al, 1993). Diacylglycerol is a second messenger, which activates protein kinase C (PKC) whereas Ins P3 stimulates the release of $Ca^{2+}$ from intracellular storage sites. Cell proliferation is an example of the response to the second messenger action. The inner side of the red cell membrane is laminated with a protein network that is mainly composed of spectrin, actin, protein 4.1 and dematin (4.9 band), so-called 'junctional complex', which stabilizes the membrane and determines functional integrity (Haest et al, 1978; Benett et al, 1990; Xiu-Li et al, 1996). Many integral membrane proteins, such as band 3 protein, glicophorin-A and -C, help maintain the regular erythrocyte membrane cytoskeleton structure (Benett, 1981; Sikorski et al, 1993*b*). Factors that affect the cells

*Received 27 August 1997*
*Revised 12 February 1998*
*Accepted 12 February 1998*

*Correspondence to:* Z Kopczyński and cause changes in the spatial structure of membrane superficial components are reviewed by Fowler (1990) and Michalak and Sarzala (1977). The aim of our studies was to quantify the changes in protein and phospholipid content of erythrocyte membranes from women with ovarian cancer.

### MATERIAL AND METHODS

### Patients

Studies were carried out on the mature membranes of erythrocytes sampled from 30 women with ovarian cancer before oophorectomy, aged 24–79 years, treated in the Gynaecology Division, Chair of Oncology, Karol Marcinkowski University of Medical Sciences in Poznań. All patients developed the third-degree clinical stage of epithelial ovarian cancer, according to UICC. Clinical diagnosis was confirmed by histopathological examination in the Department of Pathology, Chair of Oncology.

In most women in the third stage of clinical progression of the ovarian cancer a decrease in values of haemoglobin, haematocrit and erythrocytes (which are signs of anaemia) was found. Red blood cell indices in these women were within the low normal range or had a diminished value. Diminished value of mean corpuscular haemoglobin concentration and mean corpuscular haemoglobin mass could be the cause of hypoferraemia, often found in the advanced stage of cancer. Also, the erythrocyte image in the blood smear showed microcytosis traits, sometimes poikilocytosis. In some cases an increased percentage of elliptocytes up to 15% was found in the blood smear. The percentage of reticulocytes counted in the blood smear did not deviate from the normal value.

Erythrocytes from 20 healthy women, employed at the Department of Medical Analytics, Chair of Oncology, were the control samples.

### Erythrocyte membranes isolation

Venous blood samples were taken from women with ovarian cancer before surgery into 'Vacutainer' tubes pretreated with EDTA (Sarstedt, Germany).

After centrifugation at 350 ***g*** for 30 min, the plasma was collected in to separate test-tubes, then the upper layer of blood cells containing granulocytes, lymphocytes, thrombocytes and reticulocytes was removed. The remaining erythrocytes were washed twice with natrium chloride isotonic solution. The samples were then centrifuged at 350 ***g*** for 10 min and each time the upper layer of red blood cells was removed. The above procedure gave a homogeneous suspension of erythrocytes completely deprived of white blood cells, which was confirmed by microscope examination. The haematocrit value of the erythrocytes obtained was determined and the red blood cells were then haemolysed with 20 ml of cold 10 mM Tris, 2 mM EDTA buffer, pH 7.5. Next, samples were centrifuged at 2°C, 50 000 ***g*** for 10 min, the supernatant was removed and the sediment containing erythrocyte membranes was washed twice with 10 ml of the same cold buffer (Barwijuk et al, 1989).

### Phospholipids isolation

Phospholipids were isolated from membranes by the acidic extraction method (Müller et al, 1986). In two-dimensional thin-layer chromatography on the plates covered with silica gel, nine fractions of phospholipids were separated (Broekhuyse, 1969). The first dimension was developed with chloroform–methanol–6.5 mM ammonium hydroxide–water (98:74:13:8) and the second dimension involved the used of chloroform–methanol–14 mM acetic acid–water (90:13:50:10)

The separated phospholipid fractions were developed in iodine vapour then transferred with gel into tubes and 1 ml of 60% hyperchloric acid was added to each tube to mineralize the samples during 1 h of heating at 160°C, then the concentration of total phosphorus was determined (Bartlett, 1959). The obtained value was the base to quantitative evaluation of phospholipids in the separated fractions.

### Phospholipase C activity measurement

The activity of PLC was determined spectrophotometrically (Akhrem et al, 1984). PLC activity was expressed as conventional units (U $ml^{-1}$), which means the phosphorus quantity released during 30 min of phospholipid hydrolysis at 37°C to aqueous phase, in relation to the total phosphorus capacity.

Simultaneously, in the same samples, electrophoretic separation of membrane proteins was performed in ten women with ovarian cancer.

### SDS-PAGE gel electrophoresis

Red blood cell membranes from patients and healthy donors were prepared as described previously (Corningh et al, 1981; Rybczyńska et al, 1993). Briefly, haemoglobin was removed by osmotic lysis in 5 mM sodium phosphate buffer, pH 8.0 (5P8) containing phenylmethylsulfonylfluoride (PMSF) and subsequent centrifugation at 25 000 ***g*** for 10 min at 4°C, followed by washing five times with the same buffer. Protein was determined by the method of Lowry et al (1951). Red blood cell membranes were diluted to a concentration of 1 mg of protein 1 $ml^{-1}$ in SDS sample buffer (4% SDS, 10% β-mercaptoethanol, 20% glycerol, 0.005% bromophenol blue, 100 mM Tris HCl pH 6.8) and solubilized by incubation for 2 min at 100°C. Separation of membrane proteins was according to SDS-PAGE protocol performed in 7.5% polyacrylamide gel containing 0.1% SDS with 4% acrylamide stacking gel (Fairbanks et al, 1971). The proteins were stained with Coomassie brilliant blue R-250 and destained. The protein content of each band was quantified by laser densitometry (LKB Ultrascan) at 620 nm and expressed as a percentage.

### Statistical analysis

Cohran–Cox and Student's *t*-tests were used for the statistical analysis.

### RESULTS

In thin-layer two-dimensional chromatography nine fractions of phospholipids were obtained: lecithin phosphatidylcholine (PC), phosphatidylethanolamine (PE), sphingomyelin (SPH), lysophosphatidylcholine (LPC), phosphatidic acid (PA), phosphatidylinositol (Ptd Ins), phosphatidylinositol-4-phosphate (Ptd Ins-4-P), phosphatidylinositol-4,5-diphosphate (Ptd Ins-4,5-P2). Our study showed a significant decrease in phosphatidylserine (PS), SPH, LPC and PA in erythrocyte membrane of women with ovarian cancer (Figure 1). The highest reduction (of 54%) was observed for SPH, 43% for PS, 36% for LPC and 25% for phosphatidic acid concentration. However, the PE concentration in erythrocyte membranes of cancerous women did not reveal significant differences as compared with the control group.

© Cancer Research Campaign 1998

**Figure 1** The concentration of phospholipid fraction in erythrocyte membranes of women with ovarian cancer. Results presented above are means (± s.d.) of 30 independent measurements. *Statistically significant difference, $P < 0.05$. □, Control; ■, malignant

The main changes were obtained in cases of inositol-derived phospholipid fractions (Figure 2). A statistically significant threefold increase in Ptd Ins-4-P and Ptd Ins-4,5-P2 concentrations was observed with simultaneously reduced level of Ptd Ins in women with ovarian cancer.

We also found a statistically significant reduction in PLC activity in erythrocyte membrane of women with ovarian cancer. The inhibition observed here reached 71% (Figure 2).

The SDS-PAGE method allowed the following fractions to be separated: spectrin, 2.1 band, 2.2 band, 3 band, 4.1 band, 4.2 band, 5 band and 6 band (Figure 3). There are no qualitative changes in protein fractions in erythrocyte membranes of women with ovarian cancer as compared with membrane proteins of healthy women (Figure 4a and b).

However, quantitative analysis showed a decrease in spectrin level by 30% in erythrocyte membrane of cancerous patients as compared with controls. Simultaneously, a statistically significant increase in band 4.1 protein was observed (Figure 5).

### DISCUSSION

Cancer affects the entire human organism, causing biochemical changes in various cells and tissues, as well as in erythrocytes. The erythrocyte membrane is especially liable to activity in extra- and intracellular factors, which could take part in structural modification and conformation of each component (Branton, 1981). Previous studies have shown that in advanced stages of cancer changes occur in erythrocyte dimensions and shape, as well as shortening of lifespan (Miller, 1990; Honda, 1995). Several reasons for these phenomena are possible. Some authors suppose that earlier erythrocyte disintegration is a result of intensive lipid peroxidation (Roy et al, 1991). Malonyldialdehyde (MDA) is a marker for lipid peroxidation. A high concentration of MDA in the red blood cells leads to membrane 'gap' formation and raises cell susceptibility to haemolysis. Also, MDA, concentration of which is elevated in erythrocytes of cancerous patients, links amino groups of enzymes leading to a decrease in biological activity in these cells (Lubin, 1981). Another current opinion is that dinucleotide nicotinamidoadenine phosphate (NADPH) deficit is responsible for metabolic disturbances in erythrocytes of cancerous patients (Chien and Sung, 1990). NADPH is a coenzyme of glutathione and metheamoglobin reductases. A decreased level of reduced glutathione is a reason for some of the observed disturbance. These changes are due to the inhibition of activity of thiol-containing enzymes, e.g. glucose-6-phosphate dehydrogenase (E.C.1.1.1.49; Chien and Sung 1990). Many authors also suppose that modification of protein chemical composition results from the proteolytic activity

**Figure 2** The concentration of inositol-derived phospholipid fractions and phospholipase C activity in erythrocyte membranes of women with ovarian cancer. Results presented above are means (± s.d.) of 30 independent measurements. *Statistically significant difference, $P < 0.05$. □, control; ■, malignant

© Cancer Research Campaign 1998

**Figure 3** Protein composition of normal and patient red blood cell membranes. Aliquots of 10 μg each of normal membrane proteins (lane B) and patients' membrane proteins (lanes C and D) were separated on 7.5% SDS-PAGE. Lane A, SDS-PAGE molecular standard (high range)

**Figure 4** Densitometric scan of the SDS-PAGE protein separations. The peaks represent the following proteins: **A** Control, 1, spectrin; 2, band 2.1; 3, band 2.2; 4, band 3; 5, band 4.1; 6, band 4.2; 7, band 5; 8, band 6. **B** Patient, 1, spectrin; 3, band 2.1; 4, band 2.2; 5, band 3; 6, band 4.1; 7, band 4.2; 8, band 5; 9, band 6

of enzymes, expression of which on cancer cells is threefold higher than on normal cells (Michalak et al, 1977). It also seems that membrane peripheral proteins and phospholipid bilayer are responsible for the normal erythrocyte shape and osmotic resistance maintenance (Anderson and Marchesi, 1985; Hanspal and Palek, 1987). Phospholipids, as a main component of cell membrane, determine the influence of external factors on intracellular metabolism. Our studies revealed that in erythrocyte membranes from ovarian cancerous women statistically significant decreases in PS, SPH, LPC, PA and Ptd Ins occurred compared with the matched control group. Simultaneously, a statistically significant increase in phosphate derivates of inositol lipids: Ptd Ins-4-P and Ptd Ins-4,5-P2 were observed. PS concentration was significantly lower than in control subjects. Quantitative changes in erythrocyte membrane phospholipids resulting from our studies suggest assymetry disturbances, which might lead to cytoskeleton reorganization (Byers and Branton, 1985). SPH plays a fundamental role in this reconstruction because the hydrogen bonds it forms between the neighbouring molecules may regulate erythrocyte membrane condition (Schwartz et al, 1985). Another hypothesis proposes that SPH assymetric distribution is supported by the ATP-dependent system of PS translocation into inner monolayer and PS interaction with membrane cytoskeleton proteins (Vermeulen, 1996). Spectrin, as a main element of the cytoskeleton joins the membrane phospholipids by molecules of ankiryn, actin and 4.1 band protein and affects hydrophobic zone lipid bilayer settlement (Manno et al, 1995). Our densitometric analysis of erythrocyte membrane proteins showed that the spectrin level decreased by 30% and band 4.1 level increased by 27% as compared with control. These internal membrane proteins take part in the lipid transport between plasma and red blood cells and also across the bilayer membrane. They influence the removal of damaged lipid molecules (Chao and Tao, 1991; Lorenzo et al, 1994). It seems that quantitative changes in membrane protein in erythrocyte cytoskeleton may negatively influence the normal membrane organization and erythrocyte stability. Additional protein fraction of low electrophoretic mobility, which migrates more slowly than spectrin on SDS-PAGE, was found in erythrocyte membranes of ataxia-telangiectasia (A-T) patients (Rybczyńska et al, 1990). This phenomenon allows healthy individuals to be distinguished from A-T patients before the membrane defect appears. The inherited spectrin deficit may be connected to the increased red blood cell susceptibility to haemolysis–hereditary spherocytosis (Marchesi et al, 1987). A greater 4.1 band protein level may damage the chemical condition of the membrane as a consequence of unpaired interaction of 4.1 protein and myosin (Hanspal and Palek, 1987). Results received up to now have shown that PS takes part in this transformation, too. The deficit of PS in the erythrocyte membrane of women with ovarian cancer can disturb physiological rigidity of the membrane. Less elastic cell membrane reduces the osmotic resistance and shortens the erythrocyte lifespan (Rybicki et al, 1988; Pestonjamasp and Mehta, 1995). Phospholipids, particularly inositol derivates, e.g. Ptd Ins-4-P and Ptd Ins-4,5-P2, also take part in conformation changes of erythrocyte membrane proteins. Our study showed Ptd Ins-4-P and Ptd Ins-4,5-P2 accumulation, which disturbs the regular polymerization of actin fibre. This fact could be connected

© Cancer Research Campaign 1998

**Figure 5** The concentration of protein fractions in erythrocyte membranes of women with ovarian cancer. The protein content of each band was quantified by laser densitometry (LKB Ultrascan) at 520 nm and expressed as a percentage. Results presented above are means (± s.d.) of ten independent measurements. *Statistically significant difference, $P < 0.05$. □, Control; ■, malignant to the maintenance of erythrocyte physiological shape. However, Schwartz et al (1985) suggest that diacylglycerol as an intermediate phosphoinositide degradation product and a strong mediator of cell metabolism has a great significance in regular erythrocyte shape keeping (Müller et al, 1986). We observed that Ptd Ins-4-P and Ptd Ins-4,5-P2 levels were raised almost threefold in erythrocyte membranes of women with ovarian cancer compared with control subjects. High concentrations of membrane inositol lipids could have many causes. The main cause seems to be PLC inhibition. This enzyme takes part in the degradation of the phospholipid component of cell membrane. The PLC substrates are Ptd Ins-4-P and Ptd Ins-4,5-P2. Our studies showed a 70% decrease in PLC activity in erythrocyte membranes of women with ovarian cancer. High levels of Ptd Ins-4-P and Ptd Ins-4,5-P2 can be induced by intensive activity of PKC. Furthermore, an increase in Ptd Ins-4-P and Ptd Ins-4,5-P2 levels may be a consequence of stronger activity of phosphatidylinositol kinase (E.C.2.7.1.67) and phosphatidylinositol-4-phosphate kinase (E.C.2.7.1.68) (Schwartz et al, 1985). Phosphorylation processes probably dominate dephosphorylation, which is then followed by an increase in Ptd Ins-4-P and Ptd Ins-4,5-P2 levels with a simultaneous Ptd Ins reduction in concentration. The reason for the PLC inhibition in mature erythrocyte membranes in cancerous patients has not yet been sufficiently explained. The literature data reveal that a crucial role in this process may be played by an increase in quanosine diphosphate concentration, which is an inhibitor of PLC activity (Bolt et al, 1993). This enzyme can also be inhibited by pH, ionic strength of intracellular environment and reduction in calcium and magnesium intracellular concentrations. Quantitative phospholipid changes in erythrocyte membranes of cancerous women, mainly SPH, PS and inositol lipid derivates, may influence membrane cytoskeleton reorganization. Phospholipid changes are accompanied by differences in structural proteins, observed in our studies. Altogether, they may contribute to the essential erythrocyte membrane property disturbances and could influence activity and integrality of red blood cells. These quantitative changes may be associated with permanent or transient anaemia in women with ovarian cancer.

### ABBREVIATIONS

LPC, lysophosphatidylcholine; MDA, malonyldialdehyde; PA, phosphatidic acid; PC phosphatidylcholine; PS, phosphatidylserine; SPH, sphingomyelin; Ptd Ins, phosphatidylinositol; Ptd Ins-4-P, phosphatidylinositol-4-phosphate; Ptd Ins-4,5-P2, phosphatidylinositol-4,5-diphosphate; PE, phosphatidylethanolamine; PLC, phospholipase C, PKC, protein kinase C.

### REFERENCES

Akhrem AA, Litvinko NM, Kisel MA (1984) Effect of substrate organization and charge on the activity of phosphatidylinositol-specific phospholipase *C*. *Biochimica* **49**: 381–387

Anderson RA, Marchesi VT (1985) Regulation of the association of membrane skeletal protein 4.1 with glycophorin by a polyphosphoinositide. *Nature* **318**: 295–298

Bartlett GR (1959) Phosphorus assay in column chromatography. *J Biol Chem* **234**: 466–470

Barwijuk AJ, Świetochowska K, Piaścik R, Jaroszewicz K, Skrzydlewski Z (1989) Phospholipid composition of plasma and erythrocytes in the neonate. *Acta Physiol Pol* **40**: 404–410

Benett F (1981) The spectrin–actin junction of erythrocyte membrane skeleton. *Biochim Biophys Acta* **88**: 107–121

Benett V (1990) Spectrin-based membrane skeleton: a multi-potential adaptor between plasma membrane and cytoplasm. *Phys Rev* **70**: 11029–11065

Bolt MJ, Bissonnette BM, Hartmann SC, Brasitus TA (1993) Characterisation of phosphoinositide-specific phospholipase C in rat colonocyte membranes. *Biochem J* **292**: 271–276

© Cancer Research Campaign 1998

Branton D, Cohen CM, Tyler J (1981) Interaction of cytoskeletal proteins in the human erythrocyte membrane. *Cell* **24**: 24–32

Broekhuyse RM (1969) Quantitative two-dimensional thin-layer chromatography of blood phospholipids. *Clin Chim Acta* **23**: 457–460

Byers TJ, Branton D (1985) Visualization of the protein association in the erythrocyte membrane skeleton. *Proc Natl Acad Sci USA* **82**: 6153–6157

Chao TS and Tao M (1991) Modulation of protein 4.1 binding to inside-out membrane vesicles by phosphorylation. *Biochemistry* **30**: 10529–10537

Chien S, Sung LA (1990) Molecular basis of red cell membrane rheology. *Biorheology* **27**: 327–344

Corningh D, Pekala A, Shulz JR, Kuo PC (1981) Huntington disease and Tourette syndrome. Electron spin resonance of red blood cells ghosts. *Am J Hum Genet* **33**: 166–171.

Derick LK, Liu SC, Christi AH (1992) Protein immunolocalization in the spread erythrocyte membrane skeleton. *Eur J Cell Biol* **57**: 317–320

Fairbanks G, Steck TL, Wallach DFH (1971) Electrophoretic analysis of the major polypeptides of the human erythrocyte membrane. *Biochemistry* **10**: 2606–2617

Ferrel J, Huestis WH (1984) Phosphoinositide metabolism and the morphology of human erythrocytes. *J Cell Biol* **98**: 1992–2004

Fowler VM (1990) Tropomodulin: a cytoskeletal protein that binds to the end of erythrocyte tropomyosin and inhibits tropomyosin binding to actin. *J Cell Biol* **111**: 471–482

Gudi SRP, Kumar A, Bhakuni V, Gokhale SM, Gupta CM (1990) Membrane skeleton–bilayer interaction is not the major determinant of membrane phospholipid asymmetry in human erythrocytes. *Biochem Biophys Acta* **1023**: 63–72

Haest CWM, Plass G, Kamp D (1978) Spectrin as a stabilizer of the phospholipid asymmetry in the human erythrocyte membrane. *Biochim Biophys Acta* **509**: 21–32

Hanspal M and Palek J (1987) Synthesis and assembly of membrane skeletal proteins in mammalian red cell precursors. *J Cell Biol* **105**: 1417–1424

Honda K, Ishiko O, Tatsuta I, Deguchi M, Hirai K, Nakata S, Sumi T, Yasui T, Ogita S (1995) Anemia inducing substance from plasma of patients with advanced malignant neoplasm. *Cancer Res* **55**: 3623–3628

Leto T and Marchesi VT (1984) A structural model of human erythrocyte protein 4.1. *J Biol Chem* **259**: 4603–4609

Lorenzo F, Venezia N, Morle L, Baklouti F, Alloisio N, Roda L (1994) Protein 4.1 deficiency associated with an altered binding to the spectrin–actin complex of the red cell membrane skeleton. *J Clin Invest* **94**: 1651–1659

Lowry OH, Rosenbrough NJ, Farr AJ, Randal RJ (1951) Protein measurement with the folin phenol reagent. *J Biol Chem* **193**: 256–275

Lubin B, Chiu D, Bastacky J, Roelofson B, Deenen LLM (1981) Abnormalities in membrane phospholipid organization in sickled erythrocytes. *J Clin Invest* **67**: 1643–1650

Manno S, Takakuwa Y, Nagao K (1995) Modulation of erythrocyte membrane mechanical function by β-spectrin phosphorylation and dephosphorylation. *J Biol Chem* **270**: 5659–5665

Marchesi SL, Letsinger JT, Speicher DW (1987) Mutant forms of spectrin alpha-subunits in hereditary eliptocytosis. *J Clin Invest* **80**: 191–198

Michalak M and Sarzala GM (1977) Asymmetry of biological membranes [Polish text]. *Post Biochem* **23**: 523–539

Miller CB, Jones RJ, Piantadosi S (1990) Decreased erythropoietin response in patients with the anemia of cancer. *N Engl J Med* **322**: 1689–1692

Müller E, Hegewald H, Jaroszewicz K, Cumme GA, Hoppe H, Frunder H (1986) Turnover of phosphomonoester groups and compartmentation of polyphosphoinositides in human erythrocyte. *Biochem J* **235**: 775–784

Pestonjamasp KN, Mehta NG (1995) Neutral polymers elicit and antibodies to spectrin, band 4.1 protein and cytoplasic domain of band 3 protein inhibit the concanavalin A-mediated agglutination of human erythrocytes. *Biochim Biophys Acta* **1235**: 10–20

Roy G, Villar LM, Lazaro I, Gonzalez M, Bootello A, Gonzalez-Porque P (1991) Purification and properties of membrane and cytosolic phosphatidylinositol-specific phospholipase C from human spleen. *J Biol Chem* **266**: 11495–11503

Rybczyńska M, Pawlak AL, Hoffmann SK, Ignatowicz R (1990) Carriers of ataxia-telangiectasia gene display additional protein fraction and changes in the environment of SH groups in erythrocyte membrane. *Biochim Biophys Acta* **1022**: 260–264

Rybczyńska M, Feo C, Marden M, Pyoart C (1993) Abnormal rheological response of erythrocytes caused by nitroimidazoles and hyperthermia. *Int J Hyperthe* **9**: 313–323

Rybicki AC, Heath R, Lubin B, Schwartz RS (1988) Human erythrocyte protein 4.1 is a phosphatidylserine binding protein. *J Clin Invest* **81**: 255–260

Schwartz RS, Chiu DTY, Lubin B (1985) Plasma membrane phospholipid organization in human erythrocytes. *Curr Top Hemat* **5**: 63–112

Sikorski AF, Diakowski W, Kuczek M (1993*a*) Erythrocyte membrane skeleton [Polish text]. *Post Biol Kom* **20**: 93–100

Sikorski AF, Bialkowska K, Bisikirska B, Szopa J (1993*b*) Erythroid and nonerythroid spectrins – structure and functions [Polish text]. *Post Biochem* **39**: 50–60

Smith JE (1987) Erythrocyte membrane: structure, function and pathophysiology. *Vet Pathol* **24**: 471–476

Vermeulen WP, Briede JJ, Roelofsen B (1996) Manipulation of the phosphatidylethanolamine pool in the human red cell membrane affects its Mg- ATPase activity. *Mol Membrane Biol* **13**: 95–102

Xiu-Li A, Takakuwa Y, Nunomura W, Manno S, Mohandas N (1996) Modulation of band 3-ankyrin interaction by protein 4.1. *J Biol Chem*, **271**(52): 33187–33191

© Cancer Research Campaign 1998